# Patient Acceptance of Remote Scribing Powered by Google Glass in Outpatient Dermatology: Cross-Sectional Study

**DOI:** 10.2196/10762

**Published:** 2018-06-21

**Authors:** Sandra Odenheimer, Deepika Goyal, Veena Goel Jones, Ruth Rosenblum, Lam Ho, Albert S Chan

**Affiliations:** ^1^ Palo Alto Medical Foundation Sunnyvale, CA United States; ^2^ The Valley Foundation School of Nursing San Jose State University San Jose, CA United States; ^3^ Department of Nursing California State University Fresno Fresno, CA United States; ^4^ Office of Patient Experience Sutter Health Sacramento, CA United States; ^5^ Palo Alto Medical Foundation Research Institute Palo Alto, CA United States

**Keywords:** acceptance, clinician burnout, communication, Google Glass, health care provider, patient, remote scribing, trust

## Abstract

**Background:**

The ubiquitous use of electronic health records (EHRs) during medical office visits using a computer monitor and keyboard can be distracting and can disrupt patient-health care provider (HCP) nonverbal eye contact cues, which are integral to effective communication. Provider use of a remote medical scribe with face-mounted technology (FMT), such as Google Glass, may preserve patient-HCP communication dynamics in health care settings by allowing providers to maintain direct eye contact with their patients while still having access to the patient’s relevant EHR information. The medical scribe is able to chart patient encounters in real-time working in an offsite location, document the visit directly into EHR, and free HCP to focus only on the patient.

**Objective:**

The purpose of this study was to examine patient perceptions of their interactions with an HCP who used FMT with a remote medical scribe during office visits. This includes an examination of any association between patient privacy and trust in their HCP when FMT is used in the medical office setting.

**Methods:**

For this descriptive, cross-sectional study, a convenience sample of patients was recruited from an outpatient dermatology clinic in Northern California. Participants provided demographic data and completed a 12-item questionnaire to assess their familiarity, comfort, privacy, and perceptions following routine office visits with an HCP where FMT was used to document the clinical encounter. Data were analyzed using appropriate descriptive and inferential statistics.

**Results:**

Over half of the 170 study participants were female (102/170, 59.4%), 60.0% were Caucasian (102/170), 24.1% were Asian (41/170), and 88.8% were college-educated (151/170). Age ranged between 18 and 90 years (mean 50.5, SD 17.4). The majority of participants (118/170, 69.4%) were familiar with FMT, not concerned with privacy issues (132/170, 77.6%), and stated that the use of FMT did not affect their trust in their HCP (139/170, 81.8%). Moreover, participants comfortable with the use of FMT were less likely to be concerned about privacy (*P*<.001) and participants who trusted their HCP were less likely to be concerned about their HCP using Google Glass (*P*<.009). Almost one-third of them self-identified as early technology adopters (49/170, 28.8%) and 87% (148/170) preferred their HCP using FMT if it delivered better care.

**Conclusions:**

Our study findings support the patient acceptance of Google Glass use for outpatient dermatology visits. Future research should explore the use of FMT in other areas of health care and strive to include a socioeconomically diverse patient population in study samples.

## Introduction

In an effort to improve health care quality, outcomes, and reduce health care costs, the Health Information Technology for Economic and Clinical Health (HITECH) Act [1] mandated the use of electronic health records (EHRs). Across the country, EHRs are now almost ubiquitously being used to support patient care by means of clinical decision support and reduction of medical errors; however, they have been associated with unintended consequences for health care providers (HCPs). HCPs are citing increased EHR administrative tasks as a key cause of clinician burnout [2]. In fact, time log studies have demonstrated that HCPs are spending more time in performing administrative tasks such as charting and less time face-to-face with their patients [3-5]. Increased administrative burden affects providers’ work-life balance, leading to decreased physician job satisfaction and burnout [6]. Increased burnout may prompt providers to leave the field of medicine. Furthermore, the use of EHR changes the patient-provider communication dynamic by drawing HCP attention away from patients [7,8], despite evidence that direct eye contact promotes effective patient-HCP communication [9]. Poor communication between patients and HCP can also affect rapport, patient satisfaction, adherence to treatment, clinical outcomes, and patient trust [10,11], highlighting the importance and need for solutions to preserve the connection that patients need with their clinicians.

The integration of medical scribes into clinical care has been introduced as one method used to preserve patient-HCP eye contact and communication and reduce HCP workload and charting [12]. Having a scribe perform EHR documentation of the clinical visit, providers can focus their attention on the patient. In this capacity, medical scribes can be present in the examination room during the medical visit or could work remotely. Remote “virtual scribes” are also able to chart patient encounters in real-time working on or off site from a Health Insurance Portability and Accountability Act (HIPAA)–secure location, significantly reducing data entry workload that EHRs place on HCPs [13]. The addition of a virtual scribe can play a critical role in the provider–patient experience by decreasing charting and allowing the provider to fully engage with the patient and, thereby, positively impacting the patient experience [12].

Google Glass (GG), a face-mounted wearable technology, is emerging as a tool in health care settings. It has been used in several patient care areas including remote electrocardiogram interpretation [14], evaluation accuracy and triage times among paramedics in the field [15], and radiological intervention procedures [16]. Although such studies have demonstrated the value of using face-mounted technology (FMT) in health care, little is known about patient perceptions surrounding providers’ use of this technology. Prochaska and colleagues [17] surveyed patient perceptions of GG in a sample of hospitalized patients. In that sample, most respondents were unfamiliar with GG. Nearly half of respondents were concerned about privacy, but most patients were open to their providers using GG if it would improve their care.

At our organization, remote scribing via FMT was implemented to tackle the issue of burnout by connecting clinicians to remote medical scribes during patient visits and enabling real-time documentation in the EHR. Initial data has demonstrated a promising reduction in HCP EHR documentation burden. However, with the growing use of remote scribing via FMT in patient care, little empirical attention has been given to FMT and patient perceptions of FMT use in outpatient settings. The purpose of this study was to examine how the use of remote scribing with GG, by HCPs, is perceived by patients in an outpatient clinical setting.

## Methods

### Overview

This descriptive cross-sectional study was approved by the Fresno State University and Sutter Health’s Institutional Review Boards. A convenience sample of patients over 18 years of age, who could read and understand English, and who were being seen for an outpatient dermatology office visit by an HCP who used FMT were invited to participate. At the time of data collection, 4 out of 11 HCPs in the outpatient dermatology clinic used FMT.

### Study Recruitment

Participants were approached by the principal investigator (PI) after their clinic office visit and were provided with a description of the study. Interested participants were taken to a private room in the clinic where the PI or research assistant verified that the study criteria were met and informed consent was obtained. Study questionnaires were completed on computer tablets, which took 5-10 minutes. Data collection occurred over a 4-day period in September 2017.

### Measures

Participants were asked to provide general demographic data, including gender, age, race, and education level. Following this, they completed a 12-item questionnaire, which was adapted from the questionnaire developed by Prochaska and colleagues [17], with added questions regarding the level of technology and privacy. The items assessed patient familiarity, comfort, and privacy level with FMT as well as their trust, relationship, and communication with their HCP. Patient trust in HCP was rated on a 4-point Likert scale from 1 to 4 (more likely to trust provider, no change, less likely to trust provider, or I do not know). Relationship and communication with HCP was rated on a 10-point scale, from 1 (poor relationship/communication) to 10 (excellent relationship/communication). Participants were also asked to rate their level of technology adoption (innovator, early adopter, early majority, late majority, and laggard). Space was left at the end of the questionnaire for participants to add any additional comments.

**Figure 1:**
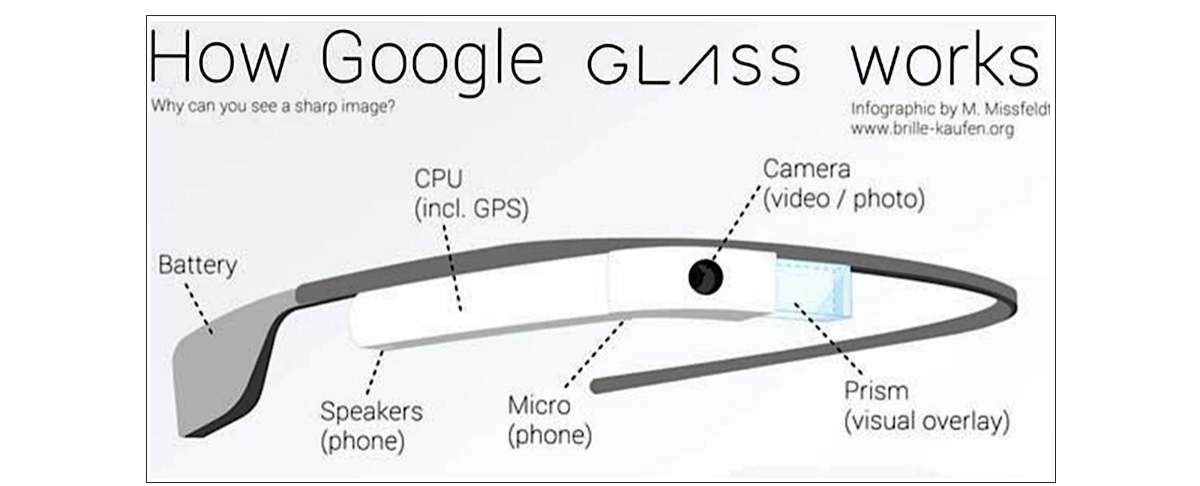
Google glass specifications. CPU: central processing unit; GPS: Global Positioning System.

### Technology and Equipment

GG is a face-mounted device that is similar to eyeglasses. The GG unit has the capability to connect to a phone via Wi-Fi enabling hands-free internet access. A small optical display is mounted above the right eye and a camera, a microphone, a speaker, and wireless connectivity is built into an eyeglass frame, which is operated through voice commands and a touchpad (see Figure 1). Each HCP in the study facility was assigned his or her own pair of GG. Custom lenses, which were compatible with GG, were available for HCPs requiring prescription glasses. Patients provided verbal permission for HCP to use GG at the beginning of each visit. In cases wherein permission was not granted, HCPs removed the GG device and remote documentation for the visit was not done.

The remote scribe observes the clinician–patient interaction and performs the documentation of the visit with HCP speaking “out loud” about the subjective history, objective findings, assessment, and treatment plan. Any comments or clarifications that the remote scribe has are communicated back via written messages that appear on the FMT display and are only visible to HCP. Remote scribes can also provide patient information within the field of vision via FMT, such as requests for data look up from the EHR, so that HCP can simultaneously perform other tasks or procedures. HCPs have the ability to receive data and input patient information through the small screen within the FMT lens, which is only discernible to the wearer, from the remote scribe. This reduces the need for HCP to look away to a desktop screen, allowing HCP to focus on the patient.

At the time of data collection for this study, HCPs had been using GG for approximately 12 months. Each HCP was assigned his or her own GG device and remote scribe. All communication between the remote scribe and HCP was encrypted and followed HIPAA operational, security, and privacy protocols to safeguard patient information. All remote scribe facilities completed a rigorous security and privacy review, which was conducted by an independent third-party auditor prior to HCP use with patients.

### Data Analysis

Descriptive data, including participant characteristics, and some questionnaire data were analyzed using descriptive statistics (frequencies, means, and measures of central tendency). Appropriate inferential statistics, including chi-square and analysis of variance tests, were used to identify the associations between variables. Narrative comments were reviewed by the research team for common themes.

## Results

### Participant Characteristics

Of the 170 participants who completed study questionnaires, over half (102/170, 59.4%) were female. The largest ethnic demographic was Caucasian (102/170, 60.0%) and second was Asian (41/170, 24.1%). The majority were college-educated or more (151/170, 88.8%). Patient ages ranged from 18 to 90 years of age (mean 50.5, SD 17.4; see Table 1).

### Patient Perception of Google Glass

#### Level of Technology

When asked to describe feelings about new technologies, 25 participants (14.7%) classified themselves as innovators (the first to adopt new technologies) and 24 (14.1%) as early majority (adopt new technology when it is still new but most people do not have it). Seventy-three (42.9%) classified themselves as early adopters (selective in adopting new technology), 41 (24.1%) rated themselves as late majority (adopt new technology after the majority of people are using it and it becomes commonplace), and 7 (4.1%) were laggards (one of the last to adopt new technology; you wait until all the bugs are out and it is inexpensive to purchase). Participants with a higher level of education were significantly more likely be among the first to adopt a new technology (X^2^_24_=64.8, *P*<.001).

When asked about their concerns surrounding the use of FMT, the majority (122/170, 73.9%) stated that they had no concerns, few (15/170, 8.8%) stated that they have security-related concerns, and very few (2/170, 1.2%) stated that FMT might be distracting.

**Table 1:** Participant characteristics (N=170).

Characteristics		Value
Age (years), mean (SD), range		50.5 (17.4), 18-90
**Gender, n (%)**		
	Female	101 (59.4)
	Male	69 (40.6)
**Ethnicity, n (%)**		
	Caucasian	102 (60.0)
	Asian	41 (24.1)
	Hispanic/Latino	12 (7.1)
	African American	4 (2.4)
	Other	11 (6.4)
**Education level, n (%)**		
	Completed high school	11 (5.9)
	Some college, no degree	16 (9.4)
	College degree	60 (35.3)
	Post graduate	75 (44.1)
	Other/prefer not to answer	9 (5.3)

#### Familiarity and Comfort With Google Glass

A large number of participants (118/170, 69.4%) reported being very or somewhat familiar with FMT, 87.1% (148/170) were extremely or somewhat comfortable with their HCP using FMT during the office visit, and 87.1% (148/170) preferred their HCP use FMT if it helped them deliver better care. Additionally, participants who were comfortable with their HCP using FMT were less likely to be concerned about privacy (X^2^_16_=89.40, *P*<.001).

#### Privacy and Trust

Few (38/170, 22.4%) participants reported being very or somewhat concerned with privacy. The majority (139/170, 81.8%) reported no change in their level of trust related to the use of FMT with 12.9% (22/170) reporting that the use of FMT would increase trust in their HCP. A significant relationship was noted between the participants’ privacy concerns relating to the use of FMT and trust in their HCP (X^2^_12_=26.5, *P*<.009).

#### Relationship and Communication

Participants’ relationships with their HCP averaged to 9.4 (SD 0.93) and communication averaged to 9.5 (SD 1.10) on 10-point scales (Table 2). Chi-square tests of independence were performed to examine the relationships between variables (see Table 2 for complete survey results).

### Participant Narrative Comments

Fifty-five participants (32.4%) provided narrative comments at the end of their surveys. Comments conveyed not noticing HCP was using FMT; for example, “I was so involved in our visit, I didn’t even notice” and “I didn’t really even notice FMT for most of the visit” as well as an overall feeling of FMT being associated with better care, “If it helps her keep track of my care, I am all for it,” “I feel more details are being documented,” and “If it helps with transcription then it is a great idea.” Comments also conveyed patient satisfaction; for example, “If it provides more face time with the doctor, I think it is worth it,” and “It is nice to have more interaction with the doctor versus them looking at the computer to take notes.”

**Table 2:** Perception of Google Glass (N=170).

Question		n (%)
**Are you familiar with Google Glass?**		
	Very familiar	16 (9.4)
	Somewhat familiar	102 (60.0)
	Neither familiar or unfamiliar	16 (9.4)
	Somewhat unfamiliar	11 (6.5)
	Very unfamiliar	25 (14.7)
**How comfortable were you when your dermatology provider was wearing Google Glass?**		
	Extremely comfortable	110 (64.7)
	Somewhat comfortable	38 (22.4)
	Neither comfortable or uncomfortable	19 (11.2)
	Somewhat uncomfortable	2 (1.2)
	Extremely uncomfortable	1 (0.6)
**Was privacy a concern when your dermatology provider was using Google Glass?**		
	Very concerned	10 (5.9)
	Somewhat concerned	28 (16.5)
	Neither concerned or unconcerned	35 (20.6)
	Somewhat unconcerned	15 (8.8)
	Very unconcerned	82 (48.2)
**How did Google Glass affect your trust in your dermatology provider?**		
	More likely to trust my provider	22 (12.9)
	No change	139 (81.8)
	Less likely to trust my provider	2 (1.2)
	I do not know	7 (4.1)
**If your dermatology provider said that Google Glass >helped them to deliver better care, would you want them to wear Google Glass during your next visit?**		
	Yes	148 (87.1)
	No	3 (1.8)
	I do not know	11 (6.5)
	I need to know more	8 (4.7)
**Would you have concerns if your dermatology provider used Google Glass during a visit? Check all that apply.**		
	I would have no concerns	122 (73.9)
	Security	15 (8.8)
	Privacy	28 (16.5)
	It may be distracting	1 (1.2)
	Unfamiliar with Google Glass	7 (4.1)
	Other: Security and Privacy	8 (4.7)
	Multiple answers	11 (6.6)
**Choose the phrase from the list below that best describes your feelings about new technologies. You are:**		
	An Innovator: First to adopt new technology	25 (14.7)
	An Early Adopter: Selective in adopting new technology	73 (42.9)
	An Early Majority: Adopt new technology when it is still new but most people do not have it	24 (14.1)
	A Late Majority: Adopt new technology after the majority of people are using it and it becomes commonplace	41 (24.1)
	A Laggard: One of the last to adopt new technology, you wait until all the bugs are out and it is inexpensive to purchase	7 (4.1)

## Discussion

### Principal Findings

To our knowledge, this is the first study to examine patient perceptions of FMT in an outpatient clinical setting; our findings build upon the work of Prochaska and colleagues [17] who examined patient perceptions of GG in a hospital setting. Although the primary reason FMT was implemented at our organization was to decrease charting documentation time and reduce clinician burnout, our results showed that FMT allowed providers to improve their interactions with patients. Remote medical scribes can alleviate HCP documentation burden and our findings support the use of virtual scribes and FMT, given that few participants expressed concern (38/170, 22.4%) with the use of FMT, 81.8% (139/170) reported no change in their level of trust with the use of FMT, and 87.1% (148/170) wanted their HCP to use FMT if it helped him or her deliver better care. Overall, our findings are in agreement with those of the study conducted by Prochaska and colleagues [17] who found that 65% (56/86) of respondents wanted their doctors to wear GG if it improved their care.

A key consideration with the deployment of remote scribes is how FMT might affect privacy and trust between the clinician and patient. Contextually, this is interesting because unlike in-person scribes that are physically present in the exam room at time of the clinical encounter, the remote scribe is not known or ever seen by the patient. In contrast to the study by Prochaska and colleagues [17], wherein nearly half of respondents were concerned about privacy, fewer participants (38/170, 22.3%) in our study were concerned with the privacy of their personal health information. Participant comments reflected little concern with trust and privacy, and the use of FMT did not change their level of trust in providers. Patients preferred their HCP use FMT ((148/170; 87.1%) if it helped deliver better care. Our institution’s deployment of remote scribing over 4 years and patient experience of the benefits versus a survey of the theoretical use of FMT administered in a hospital setting may explain the difference in concerns about privacy in our sample.

Patients in our study were more familiar with GG (118/170, 69.4%) than those (23/86, 27%) in the study conducted by Prochaska [17]. The majority of our study participants (122/170, 71.7%) considered themselves as early adopters of technology which could be explained by the fact that the study was conducted in Silicon Valley, the nation’s technology center. For many Silicon Valley residents, cutting edge technology is a part of their normal lives [18]. This familiarity and comfort may again be a consequence of an overall higher technology adoption in our geographical region. Although otherwise ethnically diverse, this study’s population was largely college-educated (151/170, 88.8%), which could also have contributed to their comfort and acceptance of the technology.

Study participants gave HCPs, who used FMT, high ratings (9.5 out of 10) regarding the perceptions of effective communication. Patients perceived that HCPs using FMT were more attentive, had greater focus, and communicated greater empathy. Also, based on patient comments, participants were more satisfied with their visits and felt that HCPs delivered more personal experiences by spending less time on the computer. This is supported by literature, which demonstrates that HCPs who spend more time communicating face-to-face, focusing their attention more on the patient and less on EHR, can positively influence their communication with patients [6]. Furthermore, patients noted that the improved interaction with their HCP made them less aware of the use of the GG technology.

The use of remote scribing via GG in outpatient dermatology settings may reduce HCP documentation time, increase efficiency, reduce charting errors/omissions, and reduce workflow stress. Health care providers can simply interact with their patient, reviewing their clinical histories and examination findings, empowered by a remote scribe who enters data directly into the patient’s EHR in real-time. This novel approach may facilitate a more meaningful use of EHR and realization of its benefits in clinical care without adding an administrative burden to HCPs. Owing to the fact that EHRs are now an integral part of clinical practice, health care organizations should continue to seek new methods of using EHR in ways that improve provider satisfaction, organizational efficiencies, and patient-provider interactions.

### Limitations

Our findings should be interpreted with caution, given the largely insured and well-educated sample, limiting generalizability to lower socioeconomic populations. Additionally, the study sample was drawn from a Northern California clinic located in Silicon Valley, where technology may be more readily accepted than in other parts of the United States. This technology acumen could account for the participants’ lack of concerns when using FMT, and it is possible that the acceptance of FMT would decrease in more rural and conservative areas. Lastly, data regarding participants’ prior encounters with HCPs at the study site were not collected, further limiting study findings.

### Conclusions

Our study findings identified a high level of patient acceptance of FMT in a dermatology clinic setting where FMT was implemented in an effort to reduce provider burnout through the use of virtual remote scribes. Future research examining wearable technology such as GG should strive to include patients from other clinical settings and from diverse geographic areas and socioeconomic backgrounds. Other outcomes, for example, HCP satisfaction with FMT, whether the use of FMT increases HCP efficiency/productivity, HCP relationships with medical scribes, and experiences of medical scribes should also be examined in future studies.

## Abbreviations

EHR: electronic health record
FMT: face-mounted technology
GG: Google Glass
HCP: health care provider
HIPAA: Health Insurance Portability and Accountability Act of 1996
HITECH: Health Information Technology for Economic and Clinical Health
PI: primary investigator

## References

[1] US Department of Health & Human Services.

[2] Bodenheimer T, Sinsky C (2014). From triple to quadruple aim: care of the patient requires care of the provider. Ann Fam Med.

[3] Arndt BG, Beasley JW, Watkinson MD, Temte JL, Tuan W, Sinsky CA, Gilchrist VJ (2017). Tethered to the EHR: Primary Care Physician Workload Assessment Using EHR Event Log Data and Time-Motion Observations. Ann Fam Med.

[4] Payne TH, Corley S, Cullen TA, Gandhi TK, Harrington L, Kuperman GJ, Mattison JE, McCallie DP, McDonald CJ, Tang PC, Tierney WM, Weaver C, Weir CR, Zaroukian MH (2015). Report of the AMIA EHR-2020 Task Force on the status and future direction of EHRs. J Am Med Inform Assoc.

[5] Tai-Seale M, Olson CW, Li J, Chan AS, Morikawa C, Durbin M, Wang W, Luft HS (2017). Electronic Health Record Logs Indicate That Physicians Split Time Evenly Between Seeing Patients And Desktop Medicine. Health Aff (Millwood).

[6] DiAngi Y, Longhurst C A, Payne T H (2016). Taming the EHR (Electronic Health Record) - There is Hope. J Fam Med.

[7] Asan O, Young HN, Chewning B, Montague E (2015). How physician electronic health record screen sharing affects patient and doctor non-verbal communication in primary care. Patient Educ Couns.

[8] Margalit RS, Roter D, Dunevant MA, Larson S, Reis S (2006). Electronic medical record use and physician-patient communication: an observational study of Israeli primary care encounters. Patient Educ Couns.

[9] Kraft-Todd GT, Reinero DA, Kelley JM, Heberlein AS, Baer L, Riess H (2017). Empathic nonverbal behavior increases ratings of both warmth and competence in a medical context. PLoS One.

[10] Shachak A, Hadas-Dayagi M, Ziv A, Reis S (2009). Primary care physicians' use of an electronic medical record system: a cognitive task analysis. J Gen Intern Med.

[11] Tabler Jennifer, Scammon Debra, Kim Jaewhan, Farrell Timothy, Tomoaia-Cotisel Andrada, Magill Michael (2014). Patient care experiences and perceptions of the patient-provider relationship: A mixed method study. Patient care experiences and perceptions of the patient-provider relationship: A mixed method study.

[12] Earls S, Savageau Judith A, Begley Susan, Saver Barry G, Sullivan Kate, Chuman Alan (2017). Can scribes boost FPs' efficiency and job satisfaction?. J Fam Pract.

[13] Brady K, Shariff Afser (2013). Virtual medical scribes: making electronic medical records work for you. J Med Pract Manage.

[14] Jeroudi OM, Christakopoulos G, Christopoulos G, Kotsia A, Kypreos MA, Rangan BV, Banerjee S, Brilakis ES (2015). Accuracy of remote electrocardiogram interpretation with the use of Google Glass technology. Am J Cardiol.

[15] Cicero MX, Walsh B, Solad Y, Whitfill T, Paesano G, Kim K, Baum CR, Cone DC (2015). Do you see what I see? Insights from using google glass for disaster telemedicine triage. Prehosp Disaster Med.

[16] Vorraber W, Voessner S, Stark G, Neubacher D, DeMello S, Bair A (2014). Medical applications of near-eye display devices: an exploratory study. Int J Surg.

[17] Prochaska M, Press V, Meltzer D, Arora V (2016). Patient Perceptions of Wearable Face-Mounted Computing Technology and the Effect on the Doctor-Patient Relationship. Appl Clin Inform.

[18] Ryan C (2017). https://census.gov.

